# Adolescents with substance use problems in outpatient treatment: a one-year prospective follow-up study focusing on mental health and gender differences

**DOI:** 10.1186/s13011-022-00482-2

**Published:** 2022-07-15

**Authors:** Karin Boson, Mats Anderberg, Johan Melander Hagborg, Peter Wennberg, Mikael Dahlberg

**Affiliations:** 1 Department of Psychology, Inland Norway University of Applied Sciences, PO box 400, NO-2418 Elverum, Norway; 2 Department of Psychology, University of Gothenburg, PO Box 500, SE-405 30 Gothenburg, Sweden; 3 Department of Social Work, Linnaeus University, P G Vejdes väg, SE-351 95 Växjö, Sweden; 4 Department of Global Public Health, Karolinska Institute, Norrbackagatan 4, SE-171 76 Stockholm, Sweden; 5 Department of Public Health Sciences, Stockholm University, SE-106 91 Stockholm, Sweden; 6 Department of Pedagogy and Learning, Linnaeus University, P G Vejdes väg, 351 95 Växjö, Sweden

**Keywords:** Adolescents, Substance use problems, Outpatient treatment, Mental health problems, Risk factors, Gender differences

## Abstract

**Background:**

Although several studies have found a high incidence of coexisting mental health problems among adolescents with substance use problems, follow-up studies addressing how these conditions change over time are rare. The study will describe and analyze indications of mental health problems and how various risk factors predict outcomes 1 year after initial treatment contact. In addition, gender-specific risk factors are explored.

**Methods:**

A clinical sample of 455 adolescents (29% girls, median age 17 years) answered a structural interview at baseline and were followed up using official records 1 year after initiated treatment. Bivariate associations and logistic regressions were conducted to analyse the links between risk factors at the individual, social, and structural levels as well as links between various mental illness symptoms at treatment start and indications of mental health problems 1 year later were analysed.

**Results:**

The results show that mental health problems among adolescents largely persisted 1 year after start of outpatient care for substance use problems. Forty-two per cent of the sample displayed indications of mental health problems at follow-up, and registrations for both outpatient treatment and psychiatric medication were more common among the girls. Girls also reported more mental illness symptoms at treatment start than boys did, especially anxiety. Depression and suicidal thoughts had predictive values regarding indications of mental health problems and small cumulative effects were found for 6–10 co-occurring risk factors.

**Conclusions:**

Adolescents with depression and suicidal thoughts at treatment start should yield attention among clinicians as these general risk factors could predict indication of mental health problems at 1 year follow-up effectively. Also, patients with more than six co-occurring risk factors seem more vulnerable for continued mental health problems. Generally, girls displayed a greater mental health and psychosocial burden at treatment initiation and were more likely to show indication of mental health problems at follow-up. These results suggests that girls are more likely to get psychiatric out-treatment parallel to, or after, substance abuse treatment. We recommend further investigation of gender differences and gender-specific needs in substance use treatment.

## Background

Mental health problems have increased dramatically among children and adolescents in Sweden over the last two decades, particularly among young women. Mental health problems are found in roughly 15% of young women, while the corresponding figure for young men is 10% [1, 2]. The increase manifests itself in the form of self-reported mental health problems, diagnosed mental illnesses, and prescriptions for psychopharmaceutical drugs. Depression, various anxiety syndromes, and neuropsychiatric diagnoses such as ADHD account for most of the increase. The Public Health Agency of Sweden [3] has pointed to poorer functioning schools and labour market changes, which create stress and psychosomatic problems, as key factors in this trend. The changes in the labor market that are mentioned are difficulties in finding work as well as precarious and temporary employment. Greater mental health problems among adolescents also correlates strongly with greater differences in socioeconomic conditions [4, 5]. A weak social position relative to one’s age peers and to societal norms that reward success and perfection also contributes to stress-related problems among upper secondary school students [6].

Adolescents with alcohol and substance use problems often exhibit a greater degree of mental health problems [7, 8]. This is a two-way correlation, as drug use can increase the risk of mental health problems [9] as well as offering a means of coping with mental health problems [10]. There are several theories regarding the causal link between mental health problems and drug problems. Some researchers believe that mental health problems generally precede problems with alcohol and drugs [11–14], while others conclude that it is difficult to determine which condition arises first [9, 15]. For example, some studies find that substance use increases the risk of mental health problems, or that the covariation can be explained by common risk factors such as negative childhood conditions, a history of abuse, and deleterious social interactions with friends [9, 10, 16–18].

Some research indicates that mental health conditions such as behavioral problems and depression appear to persist to a large extent even after substance use problems have been treated [19], while other studies show the opposite, i.e., that many mental health problems wane or vanish in many adolescents following treatment [7, 20]. Various studies have shown that all mental health problems among adolescents with substance use problems increase the risk of drop-out and relapse, resulting in poorer treatment results than in cases of substance use alone [7, 13, 15, 21–25]. Such adolescents also often receive more comprehensive treatment, such as more sessions [21, 26]. However, other studies find no differences between substance-abusing adolescents with or without mental health problems in terms of their received treatment dose [27], commitment to treatment, drop-out level, or treatment results [26–32].

One Swedish study of 156 adolescents in outpatient care intended specifically for those with alcohol and drug problems demonstrated that both substance use problems and mental health problems were largely still present among the adolescents on follow-up 1 year later [33]. Another study of just over 1200 Australian adolescents in various types of treatment programs noted a palpable decrease in general mental stress 1 year after treatment, with the initial values exhibited by the adolescents at the start of treatment having been halved [34]. In one clinical study of 50 American adolescents, the concomitant problems decreased in general among the adolescents, and to a greater extent for internalizing rather than externalizing symptoms, which largely persisted on follow-up 1 year later [35]. There are, however, studies demonstrating similar or even better results for adolescents with externalizing problems in terms of both decreased drug use and enhanced mental wellbeing among those with concomitant mental health problems than among those with substance use problems alone [25, 29, 30, 34]. Several studies have found good treatment results for the target group in general in terms of both decreased drug use and improved mental health [7, 19, 24, 28, 29, 32, 36, 37]. One study of 2900 adolescents in outpatient care in the USA considered whether a decrease in substance abuse problems had also led to a corresponding change in mental health symptoms 1 year after treatment [38]. However, the results offered weak support for such spillover effects, and adolescents who reported continued heavy drug use also had persistently high levels of both internalizing and externalizing problems. Integrated treatment in which both conditions are addressed is considered most suitable for this target group [23, 39].

### Aim

Although several studies have found a high incidence of coexisting mental health problems among adolescents with substance use problems, follow-up studies addressing how these conditions change over time are rare. Expanding our knowledge and understanding of what factors are predictive is also very important in terms of developing and improving both preventive measures and treatments.

This article presents the results of a follow-up study as part of a longitudinal project addressing mental health problems among young people with substance use problems who undergo outpatient treatment, based on data from official records. The study will describe and analyze indications of mental health problems and how various risk factors predict outcomes 1 year after initial treatment contact. More specifically, links between risk factors at the individual, social, and structural levels as well as links between various mental illness symptoms at treatment start and potential indications of mental health problems 1 year later are analyzed. In addition, gender-specific risk factors are explored.

## Methods

This study was conducted within the framework of a research project, Treatment Research on Adolescents at the Maria clinics (TRAM), the central aim of which is to study adolescents’ change trajectories regarding alcohol and drug use, mental health, and social situation, and how specific risk and protective factors affect outcomes for various groups after outpatient treatment. The project combines data from structured interviews with adolescents at intake and data from various records at follow-up 1 year after baseline. Similar strategies have been successfully used in several Swedish studies to follow up children and adolescents placed in various forms of institutional care or sentenced to custodial care or imprisonment e.g., [40–43].

### Participants

Initial naturalistic data were collected at outpatient clinics in 12 medium-sized to large cities in Sweden. These clinics, which are specialized outpatient units for young people with substance use problems, operate in cooperation with social services and the healthcare system. All outpatient clinics offer various individualized and/or manual-based treatments of alcohol and drug use problems. The average episode of care lasts four to six months.

All adolescents aged 15 years and above who initiated contact with these outpatient clinics in 2016 were invited to participate in the study; 932 individuals were informed and asked to participate in the study by the therapist in question and 469 gave consent to participate. No register data were available for 12 individuals due to incomplete civic registration numbers or migration out of Sweden, and two adolescents had died during the follow-up period. Thus, in total 455 adolescents (29% girls, median age 17 years) participating in the follow-up study are included in the actual study group. More girls than boys reported an ongoing psychiatric treatment contact (31% vs. 17%) and an ongoing psychiatric medication at baseline (31% vs. 17%).

### Non-participation

Non-response analysis shows that the study group of 455 individuals had somewhat more serious substance use problems than did the 477 individuals who opted not to participate in the study. The study group was 29% girls, while the non-response group was 22% girls; the median age was 17 years in both groups. Regarding primary drug, both groups reported similar patterns: in the study group, 76% used cannabis, 14% alcohol, and 10% other drugs; in the non-response group, 79% used cannabis, 13% alcohol, and 8% other drugs. There were significant differences in other variables related to substance use, and the study group generally had more serious substance use problems than did the non-response group in terms of more frequent substance use (51% vs. 41%), more mixed substance use (38% vs. 26%), and a larger proportion having previous substance abuse treatment (31% vs. 20%). These results differ from those of earlier follow-up studies, in which, in contrast, groups that opted not to participate often had more serious problems [44]. The differences can likely be partially explained by the somewhat larger proportion of girls – who generally have higher psychosocial loads – in the present study group see [45].

### Measures and outcomes

When the treatment process began, initial data collection began via intake interviews. The purpose of these structured interviews was to identify problems, needs, and the current situation to enable relevant assessment and the planning and delivery of treatment. The interviews were conducted by therapists at each clinic after informed consent had been obtained from the adolescent and in accordance with a manual. The interview takes about 45 minutes to administer and contained a total of 75 questions covering the following 10 aspects of life: housing and financial support, occupation, treatment history, criminality, childhood, exposure to violence, family and relationships, physical health, mental health, as well as alcohol and drug use. The interview also covered administrative matters, sociodemographic data, and ongoing treatment contacts, and concluded with several open questions. The interview method has satisfactory reliability and validity [46]. Of the aspects addressed in the interview, 10 risk factors at the structural and individual levels were defined: 1) *lack of occupation*, 2) *problems at school*, 3) *placement in foster care/residential home*, 4) *problems in childhood environment*, 5) *early age at onset of substance use*, 6) *delinquent peers*, 7) *exposure to violence*, 8) *depression*, 9) *violent behavior*, and 10) *traumatic events*. These factors were previously used in a cross-sectional study focusing on gender differences [45].

To examine mental health in greater depth, nine mental illness symptoms were analyzed at treatment start: 1) *sleeping problems*, 2) *depression*, 3) *anxiety and worry*, 4) *concentration difficulties*, 5) *aggression*, 6) *suicidal thoughts*, 7) *hallucination*, 8) *eating disorders*, and 9) *self-harming behavior*. The mental illness symptoms were screened in the structural interview, in which participants were asked if they had experienced any of the symptoms within the last 30 days.

The measures used to analyze outcomes were based on experience gained in earlier studies and provided a multifaceted and reliable picture of the adolescents’ progress (see, e.g., [43]). The outcome measure used as an indicator of mental health problems from the registers in 2017 have been categorized based on following information: psychiatric treatment in outpatient and in-patient care or medication for a mental health disorder. Data were taken from National Board of Health and Welfare’s Patient Register, National Board of Health and Welfare’s Pharmaceutical Register. Incidence in any of these registers were coded 1= “Yes, indication of mental health problems”. No incidence was coded 0 = “No indication of mental health problems”.

### Statistical analysis

Chi-square testing of independence was used to compare frequencies between girls’ and boys’ reports regarding variables indicating mental health problems at one-year follow-up (primary outcome variable), risk factors, and mental illness symptoms reported at treatment start. Effect sizes were calculated using Cramér’s V and can be interpreted as *weak* (< 0.20), *moderate* (0.20–0.39), and *relatively strong* (0.40–0.59) according to Rea and Parker [47]. Bivariate associations were calculated between risk factors, mental illness symptoms, and indications of mental health problems at one-year follow-up. Logistic regressions were used to separately describe the predictive value of the risk factors and of specific mental illness symptoms. This was done with and without controlling for gender, age, and drug use frequency (of the primary drug). In addition, separate analyses were conducted to investigate the impact of cumulative risk load. Bonferroni correction makes results with *p*-levels above 0.0007 non-significant (i.e., 0.05/76). Results are presented both with uncorrected (*p*) and corrected p-levels (*p*_adj_). SPSS 26 (2019) was used for all statistical analyses.

## Results

Prevalence regarding the outcome variable, *indications of mental health problems*, is reported in Table 1. Significantly more girls than boys were categorized as displaying indications of mental health problems at one-year follow-up (χ^2^ (1) = 12.859, *p* < 0.0005; *p*_adj_ < 0.05; Cramér’s V = 0.168). More specifically, a larger proportion of girls had outpatient psychiatric treatment contact (χ^2^ (1) = 12.293, *p* < 0.0005; *p*_adj_ < 0.05; Cramér’s V = 0.164) as well as ongoing medical treatment for mental illness (χ^2^ (1) = 10.859, *p* < 0.001; *p*_adj_ < 0.05; Cramér’s V = 0.154) at follow-up. The results indicate that 71% (*n* = 40) of the boys and 81% (*n* = 33) of the girls who reported taking psychiatric medication at treatment start displayed indications of mental health problems at follow-up. Regarding having psychiatric contact, 74% (*n* = 39) of the boys and 83% (*n* = 34) of the girls displayed indications of mental health problems at follow-up.

**Table 1:** Indications of mental health problems at one-year follow-up. The data are presented as percentages. Gender differences were tested using a Chi^2^ test (ns = not significant)

	Total (%)	Girls (%)	Boys (%)	Cramér’s V
	***N*** = 455	***n*** = 133	***n*** = 322	
**Indications of mental health problems**	42	55	37	0.168***
Outpatient treatment, psychiatry	30	41	25	0.164***
In-patient treatment, psychiatry	6	8	6	ns
Medical treatment, mental illness	31	42	26	0.154***

*p < 0.05

**p < 0.01

***p_adj_ < 0.05

Furthermore, bivariate associations and predictive values of the risk factors, with and without controlling for gender, age, and primary drug use frequency, regarding the outcome variable *indications of mental health problems* at one-year follow-up are presented in Table 2: Model 1 (χ^2^ (10) = 26.297, *p* = 0.003; *p*_adj_ = ns; Nagelkerke = 0.076) and Model 2 (χ^2^ (13) = 39.471, *p* < 0.0005; *p*_adj_ < 0.05; Nagelkerke = 0.112). *Depression* had significant predictive value regarding *indications of mental health problems*, both singly and in combination with other risk factors (Model 1), as well as with the covariates gender, age, and primary drug use frequency included (Model 2). Gender analyses also showed differences regarding *early age at onset of substance use*, i.e., girls 29% vs. boys 20% (χ^2^ (1) = 4.092, *p* = 0.043; *p*_adj_ = ns; Cramér’s V = 0.095), *exposure to violence/abuse* i.e., girls 71% vs. vs. boys 58% (χ^2^ (1) = 7.767, *p* = 0.005; *p*_adj_ = ns; Cramér’s V = 0.131), *depression*, i.e., girls 41% vs. boys 30% (χ^2^ (1) = 4.660, *p* = 0.031; *p*_adj_ = ns; Cramér’s V = 0.101) and, *traumatic events*, i.e., girls 47% vs. boys 30% (χ^2^ (1) = 12.276, *p* < 0.0005; *p*_adj_ < 0.05; Cramér’s V = 0.164).

**Table 2:** Bivariate associations and logistic regression analyses of risk factors regarding indications of mental health problems. Odds ratios and confidence intervals are presented (*n* = 455)

	Bivariate associations	Model 1	Model 2
			Full Model
	OR (95% CI)	OR (95% CI)	OR (95% CI)
1. *Lack of occupation*	1.18 (0.73–1.90)	0.90 (0.54–1.49)	1.03 (0.59–1.78)
2. *Problems at school*	1.42 (0.91–2.20)	1.16 (0.72–1.87)	1.13 (0.70–1.83)
3. *Placement in foster care/residential home*	1.68 (1.06–2.66)*	1.65 (1.01–2.68)*	1.73 (1.053–2.85)*
4. *Problems in childhood environment*	1.22 (0.83–1.78)	0.94 (0.61–1.45)	0.98 (0.63–1.52)
5. *Early age at onset of substance use*	1.01 (0.65–1.58)	0.90 (0.56–1.46)	0.81 (0.50–1.32)
6. *Delinquent peers*	0.93 (0.59–1.49)	0.87 (0.54–1.43)	0.99 (0.60–1.65)
7. *Exposure to violence/abuse*	1.36 (0.94–2.04)	1.16 (0.75–1.79)	1.13 (0.72–1.78)
8. *Depression*	2.51 (1.68–3.74)***	2.51 (1.61–3.91)***	2.55 (1.60–4.08)***
9. *Violent behavior*	1.27 (0.81–1.98)	0.95 (0.59–1.55)	0.95 (0.58–1.57)
10. *Traumatic events*	1.16 (0.79–1.72)	0.89 (0.57–1.38)	0.80 (0.51–1.27)

*p < 0.05

**p < 0.01

***p_adj_ < 0.05

*Note.* Model 1 includes risk factors 1–10 and Model 2 risk factors 1–10 but also includes age, gender, and primary drug use frequency at intake

Table 3 shows the effect of cumulative risk linked to indications of mental health problems at one-year follow-up: Model 3: χ^2^ (2) = 11.490, *p* = 0.003; *p*_adj_ = ns; Nagelkerke = 0.034), and controlled for gender, age, and primary drug use frequency in Model 4: χ^2^ (5) = 23.910, *p* < 0.0005; *p*_adj_ < 0.05; Nagelkerke = 0.069. No gender effects were found regarding the cumulative risk.

**Table 3:** Odds ratios and confidence intervals for the association between adolescent cumulative risk and indications of mental health problems at one-year follow-up (*n* = 455)

	Model 3	Model 4
		Full Model
	OR (95% CI)	OR (95% CI)
0–2 risk factors (31%), reference	1	1
3–5 risk factors (49%)	1.32 (0.85–2.05)	1.26 (0.80–1.99)
6–10 risk factors (21%)	2.48 (1.45–4.24)**	2.33 (1.31–4.15)**

**p < 0.01

***p_adj_ < 0.05

*Note.* Model 4 includes the level of cumulative risk as well as age, gender, and primary drug use frequency at intake

Concerning mental illness symptoms, gender differences were found in *sleeping problems*, *anxiety*, *suicidal thoughts*, *concentration difficulties*, *eating disorders*, and *self-harming behavior*, see Table 4.

**Table 4:** Prevalence of mental illness symptoms at treatment initiation. The data are presented as percentages. Gender differences were tested using a Chi^2^ test (ns = not significant)

Mental illness symptoms	Total	Girls	Boys	Cramer’s V
*Sleeping problems*	53	62	49	0.113*
*Depression*	34	42	30	0.110*
*Anxiety*	51	70	43	0.248***
*Concentration difficulties*	60	69	56	0.124*
*Aggression*	23	24	22	ns
*Suicidal thoughts*	8	13	7	0.097*
*Hallucinations*	6	9	5	ns
*Neuropsychiatric diagnosis*	23	25	22	ns
*Eating disorders*	9	13	7	0.106*
*Self-harming behavior*	7	13	4	0.156**

*p < 0.05

**p < 0.01

***p_adj_ < 0.05

Bivariate associations and predictive values of the mental illness symptoms at treatment start, with and without controlling for gender, age, and primary drug use frequency, regarding the outcome variable *indications of mental health problems* at one-year follow-up are presented in Table 5: Model 5 (χ^2^ (9) = 48.329, *p* < 0.0005; *p*_adj_ < 0.05; Nagelkerke = 0.141) and Model 6 (χ^2^ (12) = 58.773, *p* < 0.0005; *p*_adj_ < 0.05; Nagelkerke = 0.169).

**Table 5:** Bivariate associations and logistic regression analyses of mental illness symptoms at treatment start regarding indications of mental health problems at one-year follow-up. Odds ratios and confidence intervals are presented (*n* = 455)

	Bivariate associations	Model 5	Model 6
			Full Model
Mental illness symptoms	OR (95% CI)	OR (95% CI)	OR (95% CI)
*Sleeping problems*	2.11 (1.44–3.10)***	1.28 (0.81–2.02)	1.38 (0.86–2.20)
*Depression*	2.61 (1.74–3.90)***	1.66 (1.01–2.75)*	1.85 (1.10–3.12)*
*Anxiety*	2.50 (1.70–3.67)***	1.46 (0.89–2.39)	1.38 (0.83–2.31)
*Concentration difficulties*	1.86 (1.26–2.76)**	1.38 (0.87–2.17)	1.39 (0.87–2.20)
*Aggression*	1.31 (0.84–2.05)	0.68 (0.40–1.15)	0.67 (0.39–1.15)
*Suicidal thoughts*	4.31 (2.03–9.14)***	3.21 (1.32–7.81)*	3.59 (1.45–8.92)**
*Hallucination*s	3.66 (1.57–8.56)**	1.76 (0.70–4.46)	1.75 (0.68–4.52)
*Eating disorders*	2.32 (1.18–4.58)*	1.43 (0.68–3.02)	1.46 (0.68–3.16)
*Self-harming behavior*	2.38 (1.13–5.03)*	0.98 (0.41–2.31)	0.92 (0.38–2.21)

*p < 0.05

**p < 0.01

***p_adj_ < 0.05

*Note.* Model 5 includes all mental illness symptoms and Model 6 mental illness symptoms as well as age, gender, and primary drug use frequency at intake

## Discussion

This study found that mental health problems among adolescents largely persisted 1 year after start of outpatient care for substance use problems. Forty-two per cent of the sample displayed indications of mental health problems at one-year follow-up, and registrations for both outpatient treatment and psychiatric medication were more common among the girls. The incidences of outpatient care visits and medication were highly correlated, likely because they are often predicated on one another. The prescribing of medications also appears to have increased, as roughly one fifth of participants reported ongoing medication on enrolment, while one third were receiving medication on follow-up. One conceivable explanation for this relatively large proportion is that treatment at these specialized outpatient clinics, which is based on close collaboration between social services and the healthcare system, also creates conditions conducive to continued contact with psychiatric care. Another possible explanation is that the adolescents themselves sought psychiatric care contact to obtain adequate help and support. A third hypothesis is that it is easier to obtain help for one’s mental health once the substance use problems have been addressed.

Another study result indicates that only two of the 10 general risk factors, i.e., *placement in foster care/residential home* and *depression*, were individually predictive of continued mental health problems on one-year follow-up. *Placement in foster care/residential home* did not remain significant after Bonferroni correction. However, placement in foster care is indicative of both vulnerability and fundamental care deficiencies. Children and young people placed in social in-patient care are at considerably greater risk of experiencing mental health and social problems later in life [43]. The second predictive risk factor is *depression*, which is largely related to the outcome metric covering medication and contact with outpatient care.

Yet another key result is that negative outcomes are linked to a constellation of risk factors, rather than to any individual factor. As previous studies have shown, no single risk factor can explain ongoing mental health problems at one-year follow-up. However, the cumulative risk suggests that six or more concomitant risk factors are associated with indications of mental health problems. There is consequently an increased risk of having a mental health problem 1 year after the commencement of treatment. This cumulative effect, however, not significant after Bonferroni correction, is highly consistent with conclusions drawn in earlier studies of the relevant target group [45, 48, 49]. The fact that multiple, combined risk factors are a better predictor of mental health problems than are individual indicators could be due to both measurement–theoretical and actual causes. On one hand, one may anticipate higher validity in data containing many indicators of extensive problems, but it is also reasonable to expect that recovery and change are reasonable when problems are present in individual areas of life, while there may be greater complexity if the problems are many and extend across multiple life domains. There may also be differences in the abilities of the treating entities to help adolescents cope with various types of other life problems.

Gender differences were also found in the general risk factors between girls and boys attending outpatient treatment for substance use problems, with more girls than boys experiencing *early age at onset of substance use*, *exposure to violence*, *depression*, and *traumatic events*. Although only *traumatic events* continued to show significant effects after Bonferroni correction. Nevertheless, our study confirms previous findings that girls in substance use treatment display greater vulnerability regarding individual and social risk factors [50, 51].

Of the self-reported mental illness symptoms associated with commenced outpatient treatment, *depression* and *suicidal thoughts* were the two that most clearly predicted continued need. Depression is a condition that often has a protracted disease course and entails long-term therapy with anti-depressive medications [52]. This is particularly concerning, as earlier longitudinal studies have shown that adolescents with depression are at increased risk of both suicide and weak establishment in the labor market later in life [53]. Girls in substance use treatment also display more severe difficulties regarding mental illness symptoms at treatment start than do boys. Significant gender differences were found regarding *sleeping problems*, *anxiety*, *suicidal thoughts*, *concentration difficulties*, *eating disorders*, and *self-harming behavior*. *Anxiety* continued to show significant difference after Bonferroni correction. The pattern remained the same at one-year follow-up, with girls, to a greater extent than boys, displaying indications of mental health problems. This is consistent with earlier research showing that young women in substance use treatment report higher rates of co-occurring psychiatric problems than do young men [51, 54–56]. It has also been found that although depression is common among women in substance use treatment, it often goes unnoticed [56]. These findings might explain the higher levels of mental health problems among girls at follow-up. Furthermore, girls with experiences of trauma and abuse are vastly overrepresented in substance use treatment [57, 58].

One of the consistent results of the study is the gender differences that emerged, as the girls continued to have more severe mental health problems than did boys at follow-up. It might also indicate that the girls more than boys sought help through psychiatric outpatient treatment. Another possible explanation is that professionals refer girls to psychiatric treatment more than they do boys. No gender differences were found concerning psychiatric in-patient treatment. However, the number of participants in this clinical sample who received psychiatric in-patient treatment was high relative to national statistics, i.e., about 6% of the participants with alcohol and substance use problems received such treatment vs. approximately 1% in a general sample aged 18–24 years [2].

One common pattern observed in alcohol and drug research is that men or boys are overrepresented in substance abuse care, despite the minor gender differences in drug use typically seen in general populations [59]. This has previously been assumed to have to do with males experiencing more pronounced problems than females. This explanation has recently increasingly been reconsidered, and alternative interpretations have been offered, for example, that the overrepresentation is instead attributable to selection factors, such as the legal system being a major referrer of patients to substance abuse care [50] or that the ratio reflects the fact that men constitute the norm in this area as well [60].

### Strengths and limitations

This study is part of a research project addressing the outpatient treatment of substance-abusing adolescents in a naturalistic context, with follow-ups through official records. The results should be interpreted somewhat cautiously, as the relevant registers do not capture adolescents who do not seek help for their problems in the healthcare system. On the other hand, this type of information also entails a certain degree of overestimation, in those isolated appointments (e.g., in outpatient care) are taken to indicate mental health problems, even though the young person may only be seeking advice regarding their worries or be the subject of a diagnostic investigation whose outcome we do not know. Register data can thus indicate the need for new care for mental health problems, or the need for ongoing care in the form of, for example, follow-up support and/or medication – i.e., the indication may be viewed as both positive and negative. Combining information from structured interviews at baseline and several different sources from official records at follow-up produces reliable data and may be an innovative method for addressing the common problem of non-participation. It is also a strength of the study that the adolescents represent several outpatient clinics in different cities, contributing to greater generalizability to adolescents in outpatient care. Although the sample may be viewed as representative, it should be emphasized that it is a national sample in a Swedish context. Swedish substance abuse care is integrated and specialized, and stands out in terms of its heterogeneity, as adolescent patients have problems ranging from mild to severe. This study is based on follow-up data 1 year after enrolment and focuses on relatively short-term outcomes. Hence, further studies are needed based on long-term follow-up of this study group.

### Implications

The findings indicate a greater need for specialized psychiatric care after 1 year among adolescents in outpatient substance abuse treatment among both girls and boys. Integrated care is crucial when patients present both substance use problems and mental illness symptoms (e.g., depressive symptoms and suicidal thoughts) at treatment start. Integrated or parallel treatment in connection with concomitant problems generally enjoys strong scientific support [7, 61]. Early intervention via school health program and social and pedagogic support in school to enhance well-being and prevent serious mental health problems are especially important for favorable development [62, 63].

Patients with experience of foster care merit extra attention as their social support networks are expected to be weak and their mental health problems are generally more widespread and complex [43]. Hence, professionals are advised to pay extra attention to young patients with experience of foster/residential home care, depression, or suicidal thoughts. In addition, patients with several co-occurring individual, social, and structural risk factors probably need a more complex treatment plan.

The results on the cumulative effect have clear clinical implications regarding the importance of conducting initial mappings in connection with the treatment of substance use problems, indicating that adolescents with more serious problems should be paid particular attention in order to support more positive development. More studies on cumulative risk are needed as our results suggest an important delimitation in connection with six or more risk factors. Analyses of what combinations of risk factors are more or less risky are also recommended for future studies.

Even though there are evidence-based models for addressing trauma and substance use simultaneously, such as Seeking Safety [64], these are not widely implemented in treatment centers in Sweden. Furthermore, earlier findings indicate gender-specific barriers to entering treatment. Since women and girls seem to have different risk factors, co-occurring mental illness symptoms, and more experiences of trauma compared with men, they might have different needs in treatment. These differences might not be adequately addressed in current substance use treatments [65]. It has been found that the effect of trauma on substance use might be especially salient for girls [66]. We recommend further investigation of gender differences and gender-specific needs in substance use treatment.

## Conclusions

This study found that mental health problems among adolescents largely persisted 1 year after start of outpatient care for substance use problems, especially among girls. Adolescents with experiences of placement in foster care/residential home, depression and suicidal thoughts at treatment start should be given extra attention regarding mental health in treatment as these general risk factors could predict indication of mental health problems effectively at 1 year follow-up. Also, patients with more than six co-occurring risk factors seem more vulnerable for continued mental health problems which somewhat indicated a dose-response effect between risk factors and mental health outcomes. Generally, girls displayed a greater mental health and psychosocial burden at treatment initiation and were also more likely to show indication of mental health problems at follow-up. These results suggests that girls are more likely to receive psychiatric out-treatment parallel to, or after, substance abuse treatment. We recommend further investigation of gender differences and gender-specific needs in substance use treatment.

## Data Availability

The dataset generated and analyzed during the current study are not publicly available due to lack of consent from participants of this study, but aggregated data are available from the corresponding author on reasonable request.
